# Structure-Guided Stapling of Dimeric Conformations and Linker Engineering Enhance Thermostability and Fine-Tune Activity of Bispecific VHH Cytokine Agonists

**DOI:** 10.3390/antib14030074

**Published:** 2025-09-01

**Authors:** Raphael Trenker, Deepti Rokkam, Andrew Morin, Priyanka Balasubrahmanyam, Verenice Paredes, Ivan Cheng, Rene de Waal Malefyt, Martin Oft, Patrick Lupardus, Sandro Vivona

**Affiliations:** Department of Discovery, Pharmacology and Translational Research, Synthekine, Menlo Park, CA 94025, USA

**Keywords:** VHH, single-domain antibody, bispecific nanobody, cytokine surrogate agonist, disulfide stapling, thermostability, developability, IL-2, IL-18, immunotherapy

## Abstract

Background: Bispecific antibodies have emerged as a promising class of therapeutics, enabling simultaneous targeting of two distinct antigens. Single-domain antibodies (sdAbs) comprising camelid variable heavy chains (VHHs) provide a compact and adaptable platform for bispecific antibody design due to their small size and ease of linkage. Methods: Here we investigate structure-activity relationship of VHH-based cytokine surrogates by combining cell signaling and functional assays with x-ray crystallography and other biophysical techniques. Results: We describe crystal structures of four unique bispecific VHHs that engage and activate the cytokine receptor pairs IL-18Rα/IL-18Rβ and IL-2Rβ/IL-2Rγ. These bispecific VHH molecules, referred to as surrogate cytokine agonists (SCAs), create unique cytokine signals that can be tuned by linker engineering. Our structural analysis reveals multiple dimeric conformations for these bispecific SCAs, where the two VHH domains can interact to form a compact structure. We demonstrate that the dimeric conformation can be enforced via engineering of a non-native disulfide bond between the VHH subunits, thus enhancing molecular thermostability. Conclusion: Our findings have important implications for the design and engineering of bispecific VHHs or sdAbs, offering a novel strategy for tuning their activity and increasing their stability.

## 1. Introduction

### 1.1. Heavy Chain Single Domain Antibodies in Protein Therapeutics

Since the first FDA approval of muromonab-CD3 in 1986, the development of monoclonal antibodies (mAbs) as therapeutics has been steadily increasing over the last four decades, resulting in over two hundred drugs approved and roughly 1400 being evaluated in clinical trials today [1,2,3,4]. In the last two decades, VHH single-domain antibodies, also known as nanobodies, have emerged alongside mAbs as a new modality in the field of targeted protein therapeutics, featuring a first FDA approval in 2019 [5]. Derived from the immune system of camelids, VHHs retain the favorable properties of a monoclonal antibody, such as specificity for their target, stability, and solubility, while offering the advantage of being encoded by a single heavy chain that does not require pairing to a light chain [6,7]. This, together with a lower molecular weight (~13 KDa), makes them versatile building blocks for assembling multi-specific antibodies with various applications. Here we focus on how bispecific antibodies have been used as cytokine agonists.

### 1.2. Cytokines as Central Regulators of the Immune Response

**Figure 1:**
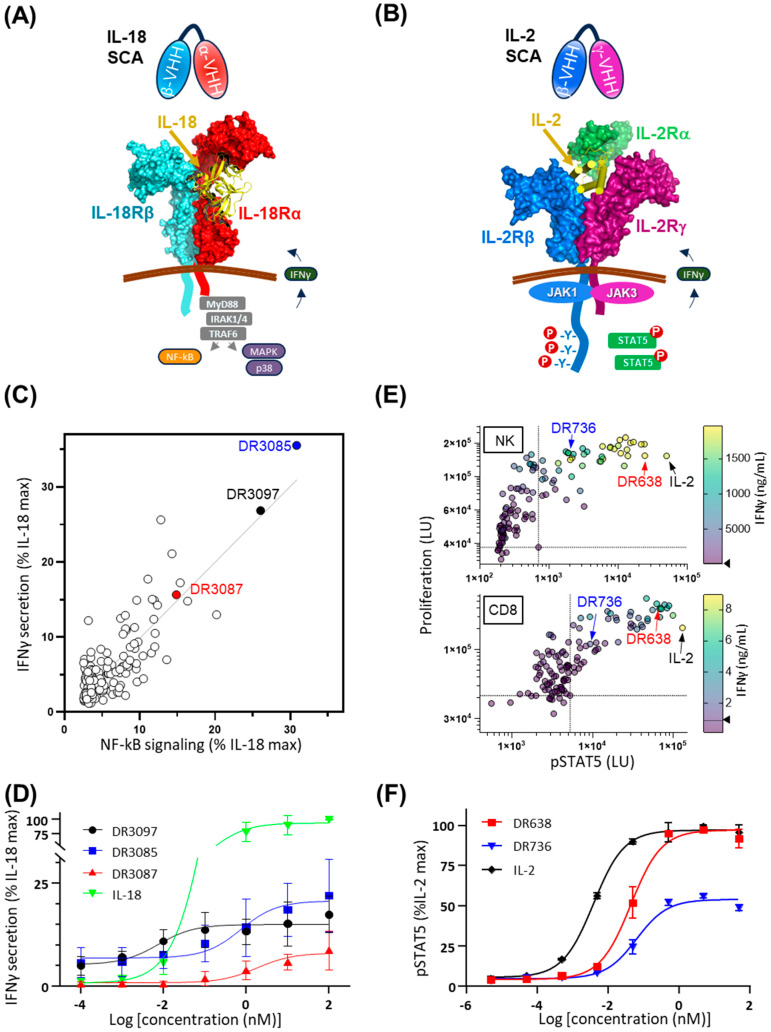
Engagement of cytokine receptors via dual, bispecific VHHs results in various levels of SCA activity. (**A**) Cartoon representation of IL-18-mediated, IL-18 receptor assembly and intracellular signaling. IL-18 SCA is composed of an anti-IL-18Rα VHH (cyan) and an anti-IL-18Rβ VHH (red). (**B**) Cartoon representation of IL-2-mediated, IL-2 ternary receptor assembly and intracellular signaling. IL-2 SCA is composed of an anti-IL-2Rβ VHH (blue) and an anti-IL-2Rγ VHH (purple). (**C**) In vitro activity of a panel of 168 IL-18 SCAs at 100 nM. The NF-kB reporter activity in a HEK-Blue NF-kB reporter cell assay is plotted against secreted IFNγ of peripheral blood mononuclear cells (PBMCs) from healthy human donors. All values are normalized as a percentage of maximal signal obtained through stimulation with IL-18. (**D**) Three IL-18 SCAs induce PBMCs to secrete variable levels of IFNγ as a function of SCA concentration, shown as a fraction of maximum secretion induced by IL-18. (**E**) STAT5 phosphorylation and proliferation of NK and CD8+ T cells isolated from peripheral blood and treated with 300 nM IL-2 SCAs and 100 pM IL-2. In the multivariate plot, each dot represents an individual VHH dimer with a color gradient referencing the amount of IFNγ secretion as shown on the scale on the right. (**F**) Two IL-2 SCAs (DR638 and DR736) specific for IL-2Rβ and IL-2Rγ receptors induce varying levels of IL-2 partial agonism, as measured via phosphorylation of STAT5 normalized to IL-2 maximum signal. Alternative text: six-panel figure (**A**–**F**). (**A**) Cartoon representation showing IL-18Rα and IL-18Rβ extracellular domains bound by IL-18 and associated into a complex on the plasma membrane. Cartoons of signaling molecules on the intracellular side lead to NF-KB activation and secretion of IFNγ, which is indicated by an arrow across the plasma membrane. A cartoon of two VHHs targeting IL-18R subunits and connected via a linker is illustrated above the receptors. (**B**) Cartoon representation of IL-2Rβ and IL-2Ry extracellular domains bound by IL-2 and associated into a complex on the plasma membrane. Cartoons of signaling molecules on the intracellular side lead to STAT5 phosphorylation and activation. A carton of two VHHs targeting IL-2 R subunits and connected via a linker is illustrated above the receptors. (**C**) A graph plotting IFNγ secretion against NF-KB signaling, both as a percentage of IL-18 maximum signal, with all 168 SCAs represented by a circle. DR3087, DR3097, and DR3085 are highlighted as strong activators of both. (**D**) A graph plotting IFNγ secretion as a percentage of IL-18 maximum signal against concentration in log scale of 3 SCAs: DR3085, DR3097, and DR3087. All SCAs show sigmoidal dose–response curves with variable midpoints (potency, EC50) and the highest maximum signal for DR3085 (20% of IL-18 maximum signal). (**E**) Two multivariate graphs plot proliferation of NK cells at the top and CD8 cells at the bottom against STAT5 phosphorylation induced by IL-2 and by 120 IL-2 SCAs, which are shown as dots. Each dot is color-coded for concentration of IFNγ in the supernatant. Two arrows point at IL-2 and DR638 in the top right corners of the plot, as they are among the most active molecules on all three parameters, while another arrow points at DR736 in the middle of the plots, as this represents a milder mimic of IL-2. (**F**) A graph plotting STAT5 phosphorylation against concentrations in log scale of two IL-2 SCAs, DR638 and DR736, as well as IL-2. All three proteins show sigmoidal dose–response curves with different midpoints and maximum values. IL-2 displays the lowest midpoint concentration (EC50) and the highest maximum signal, while DR638 and DR736 display midpoints at higher concentrations and very different maximum levels: minimal for DR736 and intermediate for DR638 compared to IL-2.

Canonical cytokine signaling is mediated by cytokine binding to two or more receptor subunits, leading to their oligomerization and induction of downstream signaling. For example, the cytokine interleukin 18 (IL-18) binds the receptor IL-18Rα and recruits IL-18Rβ to form a ternary complex, which signals through the toll/interleukin-1 receptor (TIR) domain. This in turn recruits the MyD88 adaptor protein to activate the NF-κB pathway, resulting in the release of another pro-inflammatory cytokine, interferon γ, from a broad range of cells belonging to myeloid and lymphoid lineages (Figure 1A) [8]. Similarly, the cytokine Interleukin 2 (IL-2) recruits the signaling receptor subunits IL-2Rβ and IL-2Rγ to activate JAK/STAT intracellular signaling, which is critical for the stimulation, proliferation, and growth of helper, cytotoxic, and regulatory T cells (Figure 1B).

### 1.3. Antibody-Based Cytokine Mimics

A bispecific, heavy-chain-only antibody comprising two VHHs fused to a heterodimeric Fc fragment was shown to bind the IL-2Rβ and IL-2Rγ chains and create cytokine partial agonism [9], thus mimicking the role of IL-2 in recruiting, dimerizing, and activating its receptor chains [10]. A further step forward in demonstrating the potential of VHHs for bispecific receptor engagement was achieved by the combinatorial assembly of dual VHHs into a single polypeptide chain, offering greater ease of engineering and control of agonist activity via variation in the inter-VHH linker length. Proximity-induced activation of naturally occurring receptors (IL-2/15, type I IFN and IL-10) as well as non-naturally occurring pairs (IL-2/IL-10 hybrid) proved the wide applicability of this system [11]. The advantages of antibody-based receptor dimerization over hybrid, engineered cytokine approaches [12] have become apparent, as the SCA format does not require rational design based on prior structural information and is not affected by cytokine inhibitors like IL-18 and IL-22 binding proteins [13,14]. Additionally, VHHs offer superior drug-like properties and are simpler to optimize and advance as therapeutics at both the research and development scales. For example, Lipinsky and colleagues were able to mimic IL-18 activity and circumvent IL-18’s poor drug-like properties and IL-18BP inhibition [15]. By fusing the VHH pair either at the N-terminus of an Fc or at the C-terminus of an Immunoglobulin G (IgG), the same group showed how altering the engagement geometry of the IL-12 receptor results in activity modulation and cell bias [16]. This is consistent with a previous study illustrating the power of re-orienting receptor dimer geometry in modulating cytokine signals with scFv surrogate agonists [17]. While bispecific IgG can also dimerize and agonize receptors, like the receptor tyrosine kinase MET [18], a recent report has highlighted the advantage of a narrow, compact conformation, called i-shaped antibodies, which are characterized by a Fab-Fab homotypic interaction constraining the IgG into a narrow conformation. This format has been shown to efficiently dimerize the IL-2Rβ and IL-2Rγ chains to agonize the IL-2 signaling pathway [19].

In this study, we investigate the relationship between the spatial arrangement of SCAs containing two VHHs and the resultant changes in activity. We show that modulation of signaling receptor proximity by variation in linker length and rigidity allows for tuning of cytokine signals in the IL-2 and IL-18 receptor systems. Using protein crystallography, we also discover dimeric conformations that can be locked by the insertion of an engineered disulfide bond. This stapling can significantly improve protein thermostability while retaining functional activity, thus providing a new avenue for improving the development properties of novel VHH-based drugs.

## 2. Results

### 2.1. Discovery and Characterization of IL-18Rα and IL-18Rβ Binders as Surrogate Cytokine Agonists

We adopted the screening platform [11] in which individual VHHs that target human signaling receptors IL-18Rα and IL-18Rβ were first characterized individually for receptor binding affinity, specificity, and epitope competition, followed by production as a covalently linked format on a single protein chain. This resulted in a panel of 168 dimeric SCAs derived by pairwise combination of 7 anti-IL-18Rα and 12 anti-IL-18Rβ VHHs in both orientations (A/B and B/A) and with a (G4S)2 linker in between VHHs. For in vitro functional screening, NF-kB activation was measured in a human embryonic kidney (HEK)-Blue reporter assay and by assessing interferon γ (IFNγ) secretion from PBMCs. 19 out of 168 SCAs showed NF-kB signaling and IFNγ secretion levels greater than 10% of IL-18 (Figure 1C). Three of the most active SCAs (DR3085, DR3087, and DR3097) were further characterized in a dose-response assay for IFNγ secretion. Consistent with the screening data, DR3085 drove the highest maximum IFNγ secretion, followed by DR3097 and 3087 (21%, 15%, and 8% of the IL-18 maximum signal, respectively), while EC50 values ranged from 0.01 nM of DR3097 to 0.7 and 1.8 nM of DR3085 and DR3087, respectively (Figure 1D). For reference, IL-18 maximum IFNγ secretion corresponded to 266 ng/mL with an EC50 of 0.1 nM.

### 2.2. Generation and Screening of IL-2Rβ and IL-2Rγ Surrogate Cytokine Agonists

To produce a panel of surrogate human IL-2 agonists, 120 SCAs were similarly designed by combining 10 VHHs specific for IL-2Rβ and 6 VHHs for IL-2Rγ in both sequence orientations. One signaling assay (STAT5 phosphorylation) and two functional readouts (proliferation and IFNγ secretion) were measured for both NK and CD8 cells derived from PBMC (Figure 1E). A range of activity was observed across positive hits. Although IL-2 yielded the highest pSTAT5 signaling, several SCAs yielded higher proliferation in both NK and CD8 cells and higher IFNγ secretion in NK cells. While IL-2 was used at much lower concentrations than the SCAs (100 pM for IL-2 versus 300 nM for SCAs), these results nonetheless validate that SCA-induced dimerization of the receptor chains can create potent IL-2 signals. Two SCAs of interest, one with higher potency (DR638) and a second with lower potency (DR736), are highlighted in the screen (Figure 1E). To further characterize their activity, pSTAT5 signaling was measured as a function of their concentration and compared to wild-type human IL-2, confirming that these two hits offer unique levels of IL-2 agonism (Figure 1F).

### 2.3. IL-18 and IL-2 SCA Activity Can Be Tuned via Modulation of Linker Length

**Figure 2:**
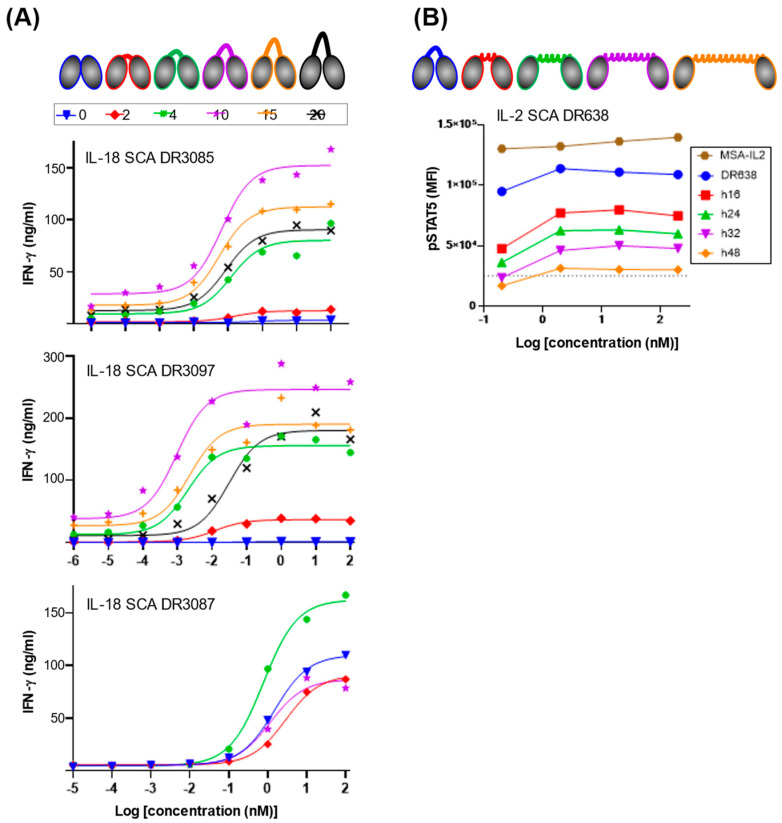
IL-18 and IL-2 SCA activity can be modulated through linker-length engineering. (**A**) DR3085, DR3097, and DR3097 IL-18 SCA partial agonism can be modulated via length variation in a flexible inter-VHH linker. The number of residues of the linker is shown color-coded at the top of the figure. IFNγ maximum levels (ng/mL) and EC50 (nM) values extracted with a log agonist vs. response fit are summarized in Table 1. (**B**) IL-2 SCA partial agonism can be modulated via length variation in a rigid inter-VHH linker. Distances between the two VHHs of DR638 were enforced by the introduction of a myosin-derived rigid alpha helix composed of 16, 24, 32, and 48 residues, which results in progressive reduction in STAT5 phosphorylation as compared to a flexible 4-residue GGGS linker (DR638) and as compared to an IL-2 fused to mouse serum albumin (MSA). Alternative text: two-panel figure (**A**,**B**). (**A**) The top of this panel shows cartoons of IL-18 SCAs connected via linkers of increasing lengths from 0 to 20 amino acids. Underneath, three separate graphs for DR3085, DR3087, and DR3097 show IFNγ secretion against SCA concentration on a log scale with sigmoidal dose–response fits of the data for each linker length. Maximum signal is obtained with linker lengths for DR3085, DR3087, and DR3097 comprising 10, 10, and 4 residues, respectively. (**B**) The top of this panel shows cartoons of IL-2 SCA DR638 connected via rigid helix linkers of increasing lengths from 16 to 48 amino acids. A graph underneath shows STAT5 phosphorylation against SCA concentration on a log scale and highlights that increasing rigid helix linker length reduces SCA activity.

It has been observed previously that altering linker length between IL-2/IL-10-targeted dual VHHs can modulate signaling through this non-natural cytokine pair [11]. To investigate modulation of activity of our three IL-18 hits by variation in the inter-VHH linker length, we initially generated a series of constructs shortening the flexible linkers from 10 residues to 4, 2, and 0 residues. For all three IL-18 SCAs, we found that linkers of less than 4 residues reduced activity (Figure 2A, Table 1). More importantly, this linker length screen revealed increased activity for one of the three SCAs, DR3087, showcasing tunability of the system through linker-length variation. The other two SCAs, DR3085 and DR3097, were screened with linker lengths up to 20 residues and showed maximal activity with the original 10-residue linker (Figure 2A, Table 1). This suggests that receptor dimerization might be distorted into a nonfunctional state when the linker is shorter than 4 residues and might be progressively hindered when the linker is extended beyond 10 residues.

To further explore the effect of linker length variation in the IL-2 SCA system, we aimed at enforcing VHH separation with a series of rigid, helical linkers consisting of fragments of a 48-residue alpha helix derived from Myosin VI [20]. We inserted 16, 24, 32, and 48 residue-long rigid helix linkers into the IL-2 SCA DR638, thus enforcing distances between the VHHs ranging from 24 to 72 Å. Compared to a 4-residue flexible linker, STAT5 phosphorylation induced by these molecules was progressively reduced as the inter-VHH distance was increased (Figure 2B), thus confirming VHH proximity can be engineered to tune SCA activity. The use of engineered rigid helix linkers offers another tool for finely tuning SCA activity.

### 2.4. SCAs Can Adopt Multiple Dimeric Conformations

**Figure 3:**
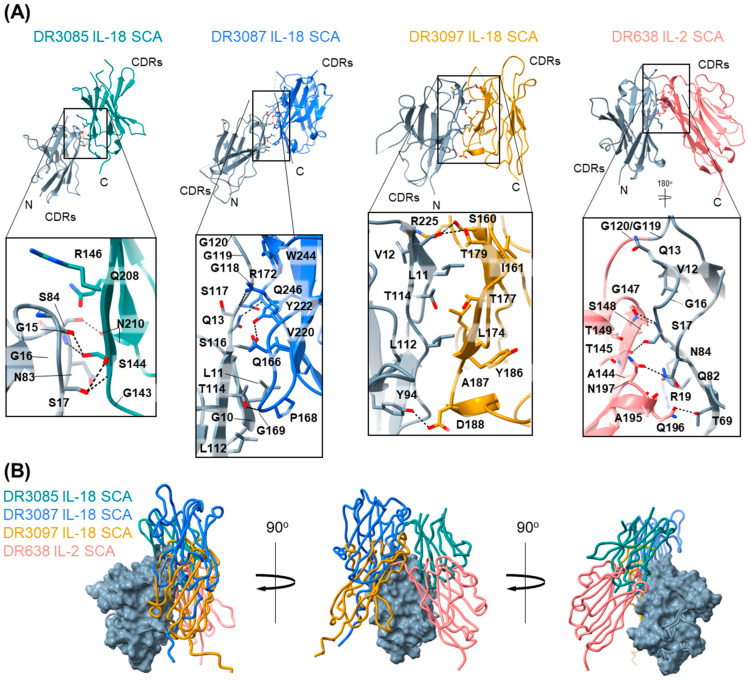
SCAs adopt dimeric conformations. (**A**) Crystal structures of four different SCAs. From left to right: DR3085 IL-18 SCA (PDB ID 9O99), DR3087 IL-18 SCA (PDB ID 9O9A), DR3097 IL-18 SCA (PDB ID 9O9B), and DR638 IL-2 SCA (PDB ID 9O9C). The N-terminal VHHs are colored gray. Residues engaged in the VHH–VHH interface (determined by ChimeraX) are shown as sticks, and hydrogen bonds are represented by dotted lines. (**B**) Overlay of crystal structures in panel A were aligned on the N-terminal VHH with the DR3087 N-terminal VHH, shown in surface representation. Alternative text: two-panel figure (**A**,**B**). (**A**) This panel shows the crystal structures of IL-18 SCAs DR3085, DR3087, DR3097, and IL-2 SCA DR638 in cartoon representation at the top, showing that the two VHHs in each SCA interact with varying degrees. Underneath each structure, a zoom-in box with all residues engaged in the interface is shown, highlighting a diverse set of hydrophobic and hydrophilic interactions. (**B**) An overlay of all four structures via the N-terminal VHH illustrates that the relative positioning of the C-terminal VHH differs between the structures. The overlay is shown from three different angles related by 90° rotations on the Y axis.

To further investigate the spatial relationship between the linked VHH in our active SCA molecules, we determined the crystal structures of three IL-18 SCAs (DR3085, DR3087, and DR3097) and one IL-2 SCA (DR638). All four SCAs crystallized in a dimeric conformation where the two linked VHHs interact to form unique antiparallel VHH–VHH interfaces with their complementary determining regions (CDRs) pointing in opposing directions in all structures (Figure 3A). Interestingly, comparison of the four structures by overlaying the N-terminal VHH shows that, while the C-terminal VHHs of each SCA cluster on the same face of the N-terminal VHH, they adopt four different inter-VHH interactions (Figure 3B). This suggests that the variable structural determinants across different VHHs contribute to different dimeric conformations with distinct interfaces.

IL-18 SCAs DR3085 and DR3087 both adopt a conformation in which the two VHHs are in an antiparallel orientation with the C-terminal VHH shifted upwards relative to the N-terminal VHH. This offset places the interaction interface towards the VHH C-terminal side and increases the distance between the CDRs. The VHH–VHH interface of DR3085 is relatively small, with a total buried surface area (BSA) of 578 Å^2^, and comprises small hydrophilic (serine, asparagine) and glycine residues that engage in sidechain-sidechain and sidechain-backbone hydrogen bonding. In DR3085, the entire linker between the VHHs is not resolved, but the C- and N-termini are about 27 Å apart, which is compatible with the 10-residue linker in this SCA and correlates with its maximal activity (Figure 3A). The DR3087 VHH interface features a more diverse set of hydrophobic and predominantly hydrophilic and ionic residues that engage in an extensive hydrogen bond network and bury a total surface area of 950 Å^2^. Out of the IL-18 SCAs evaluated, DR3097 exhibited the largest VHH–VHH interface with a BSA of 1402 Å^2^. The VHHs are positioned side-to-side against each other and engage in a large interaction network comprising hydrogen bonds and various hydrophobic residues in the center of the interface. Interestingly, many of the residues involved in and around this interface lie within the CDRs (S160, N159 in CDR1, T179 in CDR2, and G228 in CDR3) of the C-terminal, anti-IL-18Rβ VHH and are therefore unique to DR3097, underpinning the importance of these regions for SCA conformation.

The DR638 IL-2 SCA adopts a conformation that is similar to DR3097 in that the VHHs are positioned adjacent to each other in an antiparallel orientation, but in this case adopting an inverted V-shape conformation, possibly due to the linker comprising 4 residues instead of 10. As a result, VHH–VHH interactions cluster predominantly in the form of many hydrogen bonds at the top of the V-shape, resulting in a lower total BSA of 861 Å^2^. Similarly to DR3097, the DR638 C-terminal VHH CDRs are close to the VHH interface and involve a CDR residue (G147) in the interface hydrogen–bond network. Overall, our structures of four different SCAs show that they each adopt unique inter-VHH geometries with distinct VHH–VHH interfaces.

### 2.5. Engineered Disulfide Bonds Between the Two VHHs of an IL-18 SCA Result in Higher Thermostability and Different Activity Levels

**Figure 4:**
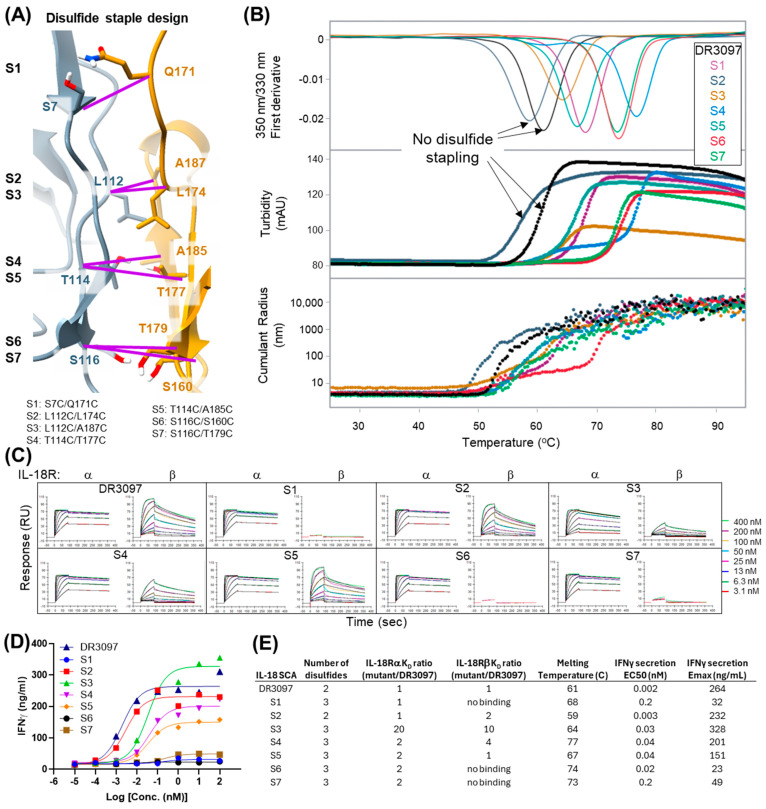
Stapling N- and C-terminal VHHs via different inter-VHH disulfide pairs results in modulation of thermostability and activity. (**A**) Residues mutated to cysteine at the interface between N- and C-terminal VHHs of DR3097 are shown and paired as outlined in purple. (**B**) Thermostability of the seven stapled SCAs measured by Nano DSF, turbidity, and particle radius as a function of temperature. (**C**) Binding of the seven stapled IL-18 SCAs to IL-18Rα and IL-18Rβ receptors measured via SPR. (**D**) Whole blood PBMC secretion of IFNγ driven by the stapled SCAs. (**E**) Summary table of data presented in this Figure. Alternative text: five-panel figure (**A**–**E**). (**A**) Residues chosen for disulfide stapling across the two VHHs are shown on the crystal structure of DR3097. Purple lines connecting two residues indicate the seven staple designs. (**B**) Graph showing results of thermal unfolding experiment of the stapled DR3097 variants. The graph has three sections showing (1) first derivative of the fluorescence signal at 350/330 nm, (2) turbidity, and (3) cumulant radius over a temperature ramp from 25 to 95 °C. Overall, the results show increased thermostability of disulfide-linked variants. (**C**) This panel shows sensograms obtained from an SPR experiment in which binding of DR3097 disulfide staple variants to IL-18Rα and IL-18Rβ was measured at different concentrations, ranging between 3.1 and 400 nM. Binding to IL-18Rα remained mostly constant, while binding to IL-18Rb was lost in three variants: S1, S6, and S7. (**D**) Graph showing dose–response curve of IFNγ secretion over SCA concentration on a log scale for DR3097 and the 7 stapled variants, highlighting strong variations in biological activity, both in terms of sigmoid midpoints and maximum values. Variants S1, S6, and S7 show the lowest maximum signals, S3 surpassing the parental molecule DR3097, while S2, S4, and S5 show variable maximum values approaching that of DR3097. (**E**) A summary table of data shown in the figure highlights S4 and S5 as stapled variants with increased thermostability and marginally reduced potency to DR3097.

**Figure 5:**
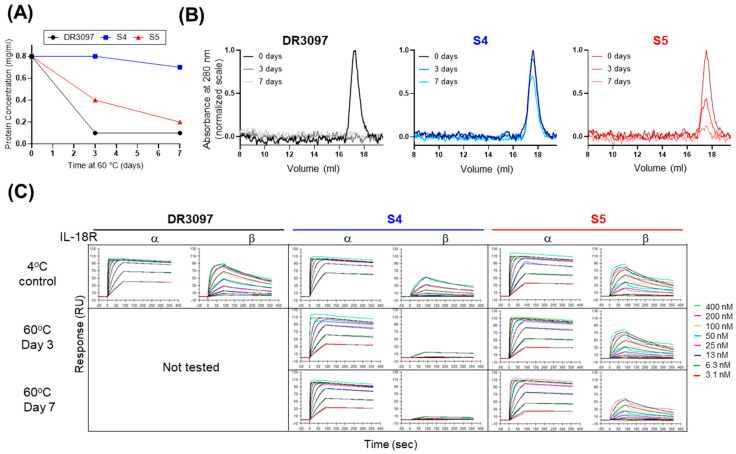
Stapled SCAs retain greater fraction of soluble protein and binding activity after temperature stress. (**A**) Protein concentration of two stapled SCAs and their non-stapled version was measured after incubation for 0, 3, or 7 days at 60 °C. Protein concentration was measured after centrifugation to remove precipitate if present. (**B**) Analytical SEC of samples in panel A using a Superdex 200 increase column in PBS. Samples were not adjusted for concentration after temperature stress. (**C**) Binding of the samples in panel A to IL-18Rα and IL-18Rβ receptors was measured via SPR. Alternative text: three-panel figure (**A**–**C**). (**A**) A graph shows protein concentration of DR3097 and variants S4 and S5 after 0, 3, and 7 days at 60 °C, with 0.8 mg/mL as the starting concentration. The concentration of S4 remains unchanged on day 3 and drops to 0.7 mg/mL only on day 7, showing the lowest propensity for precipitation and the most increased thermostability. This three-point curve contrasts with that of DR3097, which drops to 0.1 mg/mL on day 3. The S5 curve lies in between S4 and DR3097, confirming that this variant as well is more thermostable than the non-stapled control. (**B**) Three graphs showing analytical SEC elution profiles of DR3097, S4, and S5 in which absorbance at 280 nm is plotted over elution volume. Each graph shows the profiles of protein supernatant after 0, 3, and 7 days at 60 °C overlayed. All elution profiles of S4 are very similar, highlighting only a minor peak reduction at day 7 and confirming this as the most thermostable variant. In contrast, the 3- and 7-day profiles of DR3097 are flat, indicating a complete loss of monomeric fraction. S5 shows intermediate results with a notable reduction in peak height on day 3 and day 7. (**C**) This panel shows sensograms obtained from an SPR experiment in which binding of DR3097, S4, and S5 after incubation at 60 °C to IL-18Rα and IL-18Rβ was measured at different concentrations ranging between 3.1 and 400 nM SCA. S5 maintains binding to both receptors, while S4 shows reduced activity towards IL-18Rβ.

The observed dimeric conformations in our SCA crystal structures prompted questions as to whether these conformations were biologically and mechanistically relevant in situ and whether the interactions could be leveraged to increase stability or modulate activity of the construct. To enforce the dimerized conformation across the VHH–VHH interface, seven pairs of cysteine mutations (S1 to S7) were designed using the Rosetta protein design program [21] to determine structurally compatible disulfide bond positions in the DR3097 SCA (Figure 4A). The engineered (“stapled”) SCAs were produced in mammalian cells and analyzed by liquid chromatography–mass spectrometry (LCMS) to determine whether the new disulfide bond was intact. LCMS analysis showed that all stapled SCAs except for S2 formed a disulfide bond with no detectable traces of the reduced form (Supplemental File S1), suggesting that the dimeric conformation could be sampled in solution and might not be just a feature of crystal packing. Next, we investigated whether the engineered disulfides conferred a thermostability advantage to the stapled SCAs. The seven stapled SCAs, along with the original non-disulfide bonded DR3097 SCA, were subjected to a thermal melt experiment to monitor unfolding via differential scanning fluorimetry (nanoDSF), macroscopic aggregation via turbidity, and particle size via dynamic light scattering (DLS). We observed an increase in melting temperature (Tm) for all six SCAs that formed a disulfide bond over DR3097 and the S2 non-disulfide-bonded SCA. The highest thermostability improvement over DR3097 (Tm = 61 °C) was a 16 °C shift measured for S4 (Tm = 77 °C), followed by 13 °C, 12 °C, 7 °C, 6 °C, and 3 °C shifts measured for S6, S7, S1, S5, and S3, respectively (Figure 4B,E). Turbidity results were closely consistent with unfolding, suggesting that unfolding triggered macroscopic aggregation. Monitoring particle size via DLS suggests that aggregation occurs at similar onset temperatures but proceeds more readily at lower temperatures for the two non-stapled samples (Figure 4B). This data indicates that engineering a disulfide bond across the inter-VHH interfaces bestowed a thermostability advantage over the non-disulfide bonded SCAs. We next investigated whether these IL-18 SCAs would retain receptor binding affinity and receptor activation profile. Surface plasmon resonance (SPR) experiments showed that IL-18Rα affinity was retained for all variants (Figure 4C), while affinity for IL-18Rβ was lost in S1, S6, and S7. The engineered disulfides of S6 and S7 involve residues that are directly located within the anti-IL-18Rβ VHH CDRs (S160 and T179, Figure 3A and Figure 4A) and appear to be important for receptor engagement. The detrimental effect of the S1 cysteine crosslink is more difficult to reconcile, as it may have affected folding of the CDR loops specific for IL-18Rβ. Consistent with the loss of IL-18Rβ binding, S1, S6, and S7 did not drive significant IFNγ secretion. However, S2, S3, S4, and S5 showed variable levels of IL-18 activity compared to the parent molecule, DR3097 (Figure 4D,E). Given that S4 and S5 are both active and show a significant increase in thermostability, we tested whether these two molecules could indeed show improved molecular stability under prolonged isothermal stress. Therefore, we subjected S4, S5, and the DR3097 control to a 60 °C thermal hold for both 3 and 7 days, after which we assessed biophysical stability and activity. Protein concentration measurements by UV spectroscopy showed that the soluble fraction of DR3097 was reduced to 13% on day 3 and day 7, while S4 remained unaltered on day 3, consistent with no visible precipitation, and was only reduced to 88% on day 7. S5 showed intermediate results, with day 3 and 7 measuring 50% and 25% of the original concentration, respectively (Figure 5A). Consistent with the concentration measurements, integration of size exclusion chromatography (SEC) peaks showed an undetectable DR3097 monomeric fraction at day 3 and day 7. In contrast, S4 retained 92% and 70% of the monomeric peak at day 3 and 7, respectively, while S5 exhibited intermediate loss of monomeric peak with 42% and 9% at day 3 and 7, respectively (Figure 5B). We then asked whether the residual soluble fractions for S4 and S5 obtained at days 3 and 7 were active by SPR. Results indicated that binding to IL-18Rα was partially retained for S4 and largely unaffected for S5.

## 3. Discussion

Single-domain heavy-chain antibodies from camelids are desirable for use as biologics as they retain the high affinity and specificity of canonical IgG antibody formats while showing higher stability, potentially lower immunogenicity risks, and greater amenability to the assembly of multi-specific, multi-valent molecules [6,7,22]. These features have made dual VHHs, paired via linker or via Fc heterodimerization, ideal tools for creating SCAs, which require the proximal recruitment of two signaling receptor chains. Indeed, the use of VHH in dimerization of receptors has been reported for a number of signaling pairs from different receptor families, highlighting the broad applicability of this approach in generating novel cytokine agonists [9,11,15,16]. The importance of proximity when combining two VHHs into an SCA, controlled by linker length, has been investigated previously in the unnatural IL-2/IL-10 system by Garcia and colleagues, who showed that linker length modulation can be a powerful tool to fine-tune SCA-induced signaling. In this case, any extension of the linker between their IL-2/IL-10 VHHs progressively reduced STAT5 signaling [11]. Similarly, in our IL-2 system, the use of static helix linkers derived from Myosin VI to enforce spatial separation between VHHs also allows for tunability, where increased separation also led to reduced activity (Figure 2B). Observations for the IL-18 receptor system using flexible GS linkers of variable length further support the tunability of SCA activity through linker length variation. While DR3087 had maximal activity with a linker length of 4 residues, DR3085 and DR3097 showed the strongest activity in vitro with a linker length of 10 residues (Figure 2A), which was the linker length of the initial library screen. The importance of SCA connectivity and the format used by which VHHs achieve receptor oligomerization has also been observed in a previous study featuring IL-18 bispecific sdAb molecules. Rather than screening for linker length variations, this study found significant functional differences between constant sets of VHHs fused to Fc with varying paratope valencies and spatial orientations [15].

In this study we also show that SCAs can adopt distinct dimeric conformations, as highlighted by four crystal structures (Figure 3). While noncanonical dimerization of other antibody domains, for example, of IgG heavy chains, has been reported [23], this is the first crystallographic report of intermolecular assembly between camelid single-domain antibodies. All four structures described here share an antiparallel arrangement of VHHs with CDRs pointing in near opposite directions. In the four crystal structures we report, each intramolecular dimeric conformation utilizes a different dimerization interface. Together with the observation that the monomeric VHHs in solution did not show a propensity to oligomerize at concentrations up to 7 mg/mL (Supplemental File S2), this suggested that these observed interactions may be transient, aided by the intermolecular linker and dependent on the specific variable regions (frameworks and CDRs) of each VHH pair. Yet, in the case of the IL-18 SCA DR3097, the dimeric interaction has enough temporal stability in solution to ensure stoichiometric formation of the engineered disulfide bonds, as we have shown for the six DR3097 engineered disulfide pairs (Figure 4). More biophysical studies in solutions are needed to establish how frequently these interactions are sampled outside the crystallographic constraints. While we chose to engineer DR3097 due to its extensive inter-VHH interface, we hypothesize that the other three SCAs could be disulfide engineered through a similar approach. While others have shown that conformation stapling can lead to increased agonism in canonical antibodies [24], here we are not able to achieve an increase in activity beyond conferring an increase in thermostability of up to 16 °C. This is likely driven by a reduction in affinity to IL-18Rβ, due to the corresponding VHH CDR being proximal to the dimer interface. We cannot exclude that an additional effect might be contributed by structural restraint and rigidification imposed by the disulfide. Similarly, a previous study has shown how engineering two disulfide bonds between the scFv linker and the two variable domains results in reduced molecular breathing and increased thermostability without modifying activity [25]. Our work suggests that the lesser sequence similarity across the surface of VHH molecules compared to VH/VL will require evaluation of multiple designs to identify the variants offering the ideal combination of thermostability increase and potency tuning (Figure 4 and Figure 5). However, the importance of thermal stability in the drug-like properties of a candidate biologic [26,27] may make this strategy an important engineering tool for future VHH-based multispecific development.

## 4. Conclusions

Given the success of using dual, bispecific VHHs to mimic cytokines and overcome their developability and pharmacology limitations, here we provide additional insights into their linker-based tunability and propose a new engineering opportunity whereby discovery of dimeric crystal structures can be leveraged to introduce thermostabilizing disulfide bonds.

## 5. Materials and Methods

### 5.1. Immunization of Llamas (IL-18 VHHs)

Llamas were immunized with recombinant IL-18Rα extracellular domain (ECD, Met 1-Arg 329, also called IL-18R1) and IL-18Rβ ECD (Phe 20-Gly 357, also called IL-18R2 and IL-18RAP) proteins purchased from Sino Biologicals (11102-H08H, 10176-H08H). For the primary immunization, llamas received recombinant IL-18Rα and recombinant IL-18Rβ emulsified in Complete Freund’s Adjuvant (CFA). This was followed by four booster injections at two-week intervals with recombinant protein emulsified in Incomplete Freund’s Adjuvant (IFA). Blood samples were collected at three timepoints to assess the antibody response.

### 5.2. Library Construction (IL-18 VHHs)

Total RNA was extracted from PBMCs of immunized llamas using RNeasy Plus (Qiagen) according to the manufacturer’s instructions. cDNA was synthesized from RNA using reverse transcriptase and oligo(dT) primers. The variable regions of the heavy-chain-only antibodies were amplified by PCR using llama-specific primers. The PCR products were purified and digested with appropriate restriction enzymes before being ligated into a phagemid vector pADL23c. The ligation mixture was transformed into Escherichia coli TG1 cells by electroporation, and the transformed cells were plated on 2YT agar plates containing ampicillin and glucose. The library size was estimated by counting the number of colonies.

### 5.3. Phage Display (IL-18 VHHs)

The VHH phage display library was panned against recombinant IL-18Rα and IL-18Rβ to isolate specific binders. Phage particles were prepared by infecting the E. coli TG1 cells containing the library with M13KO7 helper phage. The phage particles were precipitated from the culture supernatant using polyethylene glycol (PEG)/NaCl and resuspended in phosphate-buffered saline (PBS). Three rounds of biopanning were performed. In each round, plates were coated with recombinant IL-18Rα or IL-18Rβ in PBS. After washing with PBS and blocking with 2% skim milk in PBS, the phage library was added to the plates and incubated for 1 h at room temperature. The plates were washed extensively with PBS containing 0.1% Tween-20 to remove non-specific binders. Bound phages were eluted with 100 mM triethylamine, neutralized with 1 M Tris-HCl (pH 7.4), and used to infect E. coli TG1 cells for amplification. Phage ELISA was performed to identify individual clones that specifically bound to IL-18Rα or IL-18Rβ. Positive clones were sequenced to determine the unique VHH sequences.

### 5.4. Immunization of Camels (IL-2 VHHs)

Camels were immunized with recombinant IL-2Rβ ECD (Met 1-Asp 239) and IL-2Rγ ECD (Met 1-Asn 254) proteins containing an hIgG1 Fc at the C-terminus (Novamab, Shanghai, China). Camels were acclimated for at least 7 days before immunization. The antigen was emulsified with CFA for the first injection and IFA for subsequent injections. Immunization was conducted weekly for 7 weeks, and blood samples were collected three days after the final immunization.

### 5.5. Library Construction (IL-2 VHHs)

Total RNA was extracted from PBMCs of immunized camels using standard protocols. cDNA was synthesized from the RNA using reverse transcriptase and oligo(dT) primers. The variable regions of the heavy-chain-only antibodies were amplified by PCR using camel-specific primers. The PCR products were purified and digested with appropriate restriction enzymes before being ligated into a phagemid vector pMECS. The ligation mixture was transformed into Escherichia coli TG1 cells by electroporation, and the transformed cells were plated on 2YT agar plates containing ampicillin and glucose. The library size was estimated by counting the number of colonies.

### 5.6. Phage Display (IL-2 VHHs)

The VHH phage display library was panned against recombinant ECDs of IL-2Rβ and IL-2Rγ to isolate specific binders. Phage particles were prepared by infecting the E. coli TG1 cells containing the library with M13KO7 helper phage. The phage particles were precipitated from the culture supernatant using polyethylene glycol (PEG)/NaCl and resuspended in PBS. Three rounds of biopanning were performed. In each round, plates were coated with recombinant IL-2Rβ or IL-2Rγ ECDs in PBS. After washing with PBS and blocking with 2% skim milk in PBS, the phage library was added to the plates and incubated for 1 h at room temperature. The plates were washed extensively with PBS containing 0.1% Tween-20 to remove non-specific binders. Bound phages were eluted with 100 mM triethylamine, neutralized with 1 M Tris-HCl (pH 7.4), and used to infect E. coli TG1 cells for amplification. Periplasmic extract ELISA was performed to identify individual clones that specifically bound to IL-2Rβ or IL-2Rγ. Positive clones were sequenced to determine the unique VHH sequences.

### 5.7. Protein Design, Expression, and Purification

Human IL-2 and IL-18 were purchased from Shenandoah (500-01) and Sino Biological (10119-HNCE), respectively. IL-18Rα and IL-18Rβ ECDs fused to human Fc for SPR experiments were purchased from Sino Biological (11102-H02H, 10176-H02H). Other proteins were expressed via transfection of CMV promoter vectors into Expi293 cells treated with ExpiFectamine™ according to the manufacturer’s instructions (Thermo Fisher Scientific, Waltham, MA, USA). MSA-IL-2 and SCAs were purified from Expi293 culture supernatant via C-terminal His-tag using Ni Sepharose™ Excel resin (Cytiva, Marlborough, MA, USA). Resin was equilibrated, washed, and eluted with 2, 5, and 400 mM imidazole, respectively, added to PBS and 300 mM NaCl. Proteins were buffer exchanged into PBS. For crystallization experiments, PBS was replaced with HEPES-buffered saline (HBS, 20 mM HEPES pH 7.5, 150 mM NaCl) at every step, and SCAs were additionally purified by Size Exclusion Chromatography via a Superdex 200 increase 10/300 GL column.

To screen flexible linker lengths in IL-18 SCAs, 0, 2, 4, 10, 15, and 20 amino acid GS-linkers were introduced between VHHs. To test the effect of rigid helix linkers of various lengths in IL-2 VHHs on signaling, helical linkers consisting of fragments of a 48-residue alpha helix derived from Myosin VI [20] were introduced between IL-2 VHHs (Sequence QEEEAERLRRIQEEMEKERKRREEDEQRRRKEEEERRMKLEMEAKRKQEGGQVQLQES).

### 5.8. IL-18 SCA HEK-Blue Reporter Assay

Screening of IL-18 SCA activity was performed using the HEK-Blue™ IL-18 reporter cell line (InvivoGen, San Diego, CA, USA) generated by the manufacturer by stable transfection of HEK293-derived cells with genes encoding human IL-18Rα and IL-18Rβ. In this system, IL-18-induced NF-kB activation is reported by secreted embryonic alkaline phosphatase (SEAP), which can be measured in the supernatants by the QUANTI-Blue™ assay (InvivoGen, San Diego, CA, USA). The TNFα and IL-1β signaling pathways are blocked to enable additional IL-18 specificity in this reporter assay. Wild-type (WT) IL-18 or a panel of 168 IL-18 SCAs was tested at 5.5 nM and 100 nM concentrations with the reporter assay as per the manufacturer’s instructions, respectively. Absorbance data at 630 nm quantifying SEAP activity was acquired on a BioTek Cytation 5 Plate Reader (Agilent Technologies, Santa Clara, CA, USA). Data were graphed using GraphPad Prism 10.

### 5.9. IL-18 SCA IFNγ Secretion Assay

PBMC were isolated from healthy human donors, collected in leukocyte reduction system chambers, using Lymphoprep™ (Stemcell Technologies, Vancouver, BC, Canada, Catalog #07851) and SepMate™ tubes (Stemcell Technologies, Vancouver, BC, Canada, Catalog #85450). The purified PBMCs (2 × 10^5^ cells/well in a 96-well flat-bottom tissue-culture plate) were treated with IL-18 SCAs at concentrations ranging from 0.1 fM to 100 nM (three donors) for concentration titrations or at 100 nM for single concentration screening (single donor). Human IL-12 was added at 10 ng/mL for all treatments (Peprotech, Thermo Fisher Scientific, Waltham, MA, USA, Catalog #200-12H). After 48 h of treatment at 37 °C, the supernatants were collected, and secreted human IFNγ was measured by Meso Scale Discovery (MSD, 151QOD). Data were analyzed as per the kit manufacturer’s instructions and graphed using GraphPad Prism. The SCA concentration titration displays means and standard deviations of three different donors, which were fit with a log(agonist) vs. response (three parameters) model.

### 5.10. IL-2 SCA STAT5 Phosphorylation, Proliferation, and IFNγ Production on NK and CD8 T Cells

PBMC were isolated from human Buffy Coats or Leucocyte Reduction System Chambers (LRSC) obtained from the Stanford Blood Bank using the Custom Sedimentation Kit (Miltenyi, Bergisch-Gladbach, Germany, #130-126-357) and Custom Buffy Coat/LRSC PBMC Isolation kits (Miltenyi, 130-126-448) using protocol Cust5 on an autoMACS Pro Separator (Miltenyi) according to the manufacturer’s instructions. NK cells were isolated from human PBMC using CD56 microbeads (Miltenyi, 130-050-401) on an autoMACS Pro Separator (Miltenyi) with protocol possel according to manufacturer’s instructions. PBMCs were cultured in Yssel’s medium (Iscove’s modified Dulbecco’s Medium (Thermo Fisher Scientific, Waltham, MA, USA), 0.25% w/v percent human albumin (MilliporeSigma, Darmstadt, Germany), 1% penicillin/streptomycin (Thermo Fisher Scientific, Waltham, MA, USA), 1% ITS-X Insulin, Transferrin, Selenium (Gibco, Thermo Fisher Scientific, Waltham, MA, USA), 30 mg/L Transferrin (Roche, MilliporeSigma, Darmstadt, Germany), 2 mg/L Palmitic Acid (MilliporeSigma, Darmstadt, Germany), 1% LA-OA-Albumin Linoleic Acid, Oleic Acid (MilliporeSigma, Darmstadt, Germany), and 1% human serum (GeminiBio, West Sacramento, CA, USA) at 10^6^ cells per mL with 1 μg/mL anti-CD3 mAb OKT3 (BioXcell, Lebanon, NH, USA) and 1 μg/mL anti-CD28 mAb CD28.2 (BioXcell) in 100 mL in a T150 cell culture flask (Falcon, Thermo Fisher Scientific, Waltham, MA, USA) at 37 °C for 5 days. Primary CD8 positive T cell blasts were isolated from these activated human PBMCs using CD8 microbeads (Miltenyi, 130-045-201) on an autoMACS Pro Separator (Miltenyi) with protocol possel according to manufacturer’s instructions.

For pSTAT5 induction, NK or CD8 T cell blasts were seeded into 96-well plates at 10^5^ cells per well in 95 μL DPBS prewarmed at 37 °C. Five μL of each of the 120 purified VHH dimers in DPBS was added to the cells to a final concentration of 300 nM, and plates were incubated at 37 °C for 20 min. Subsequently, 100 μL of 2× Complete Lysis buffer (Tris Lysis Buffer, Protease Inhibitor Solution, Phosphatase Inhibitor I, and Phosphatase Inhibitor II) was added according to the manufacturer’s instructions (MSD Phospho-STAT Panel K15202D). Plates were incubated on ice for 15 min and centrifuged for 5 min at 600× *g*. Lysates were transferred to a new 96-well plate. The level of phospho-STAT5 induction in the lysate was measured using the MSD multi-spot assay system with the Phospho-STAT panel kit (K15202D) according to the manufacturer’s instructions. MSD 96-well assay plates were washed 3 times with 1×Tris wash buffer, and 150 μL of Blocker A solution was added to each well. Plates were incubated on an orbital shaker for 60 min at RT and washed 3 times with 1×Tris wash buffer. Cell lysates (25 μL) were added to the plate and incubated on an orbital shaker for 60 min at RT and washed 3 times with 1×Tris wash buffer. Detection antibody solution (25 μL) was added to the plate and again incubated on an orbital shaker for 60 min at RT and washed 3 times with 1×Tris wash buffer; 150 μL of 1× Read Buffer T was added to each well, and emitted light intensity was read in luminescence units on an MSD Quickplex SQ120 instrument.

For assessment of proliferation and IFN-γ production, NK cells or CD8 T cell blasts were seeded into 96-well plates (Falcon) at 10^5^ cells per well in 190 μL in Yssels Medium. Ten μL of each of the 120 purified VHH dimers in DPBS was added to the cells to a final concentration of 300 nM, and plates were incubated at 37 °C for 72 h. 100 μL of the cell culture supernatants was transferred to a new 96 well plate for measurement of IFN-γ levels. Proliferation was measured by the Cell Titerglo assay (Promega, Madison, WI, USA). Cells were lysed by adding 100 μL per well of Cell Titerglo buffer, mixed on an orbital shaker for 2 min at 200 rpm, then held at RT for 10 min. Luminescence for cell lysates was read as counts per second in an Envision 2103 Multilabel Plate Reader (Perkin Elmer, Shelton, CT, USA).

The level of IFN-γ in the supernatants was measured using the MSD multi-spot assay system with the V-PLEX human IFN-γ kit (K151QOD-4) according to the manufacturer’s instructions. MSD 96-well assay plates were washed 3 times with 1×Tris wash buffer, and 50 μL of appropriately diluted culture supernatants were added to each well. Plates were incubated on an orbital shaker for 120 at RT and washed 3 times with 1×Tris wash buffer. Detection antibody solution (25 μL) was added to the plate, incubated on an orbital shaker for 120 min at RT and washed 3 times with 1×Tris wash buffer; 150 μL of 2× Read Buffer T was added to each well, and emitted light intensity was read in luminescence units on an MSD Quickplex SQ120 instrument.

Similarly to the pSTAT5 assay in NK cells and CD8+ T cells described above, pre-activated whole PBMCs were also used to measure IL-2 SCA activity as a function of SCA concentration. Briefly, two samples of freshly isolated, healthy donor PBMCs were activated for 72 h using the human T cell activation and expansion kit (Miltenyi) as per the manufacturer’s instructions with 1:1 cells to activation beads and 100 pM human IL-2 (Peprotech, Thermo-Fisher Scientific, Waltham, MA, USA) in Yssel’s medium. After 72 h, the activated PBMCs were washed, rested for 2 hrs, and placed in a 96-well assay plate in PBS supplemented with 2% FBS at 1 × 10^5^ cells/well. The cells were treated with 8 concentrations of IL-2 SCAs ranging from 50 nM down to 5 fM (10-fold serial dilutions) for 20 min at 37^0^C. Then MSD Tris lysis buffer was added for pSTAT5 detection by MSD (K150IGD) as per the manufacturer’s instructions and as described above. Data was plotted as mean and standard deviations of duplicates in GraphPad Prism.

### 5.11. IL-2 SCA Linker Variant STAT5 Phosphorylation on Total T Cells

Healthy human donor T-cells were thawed and stimulated (1:1) with CD3/CD28 T-cell activation beads (Gibco, 1132D) at 1e6 cells/mL of RPMI-10% FBS media supplemented with 1 nM Human IL-2 (Peprotech, 200-02-1MG) for 24 h. CD3/CD28 activation beads were removed magnetically, and cells were washed 3 times with a total of 3 volumes of non-IL-2-supplemented RPMI-10% FBS media before being re-seeded at 1e6 cells/mL and allowed to rest overnight without further stimulation. Following an 18 h rest, T-cells were stained for viability and distributed in individual wells at 3 × 10^5^ viable cells per 100 μL in PBS+2% FBS and warmed to 37 °C. IL-2 SCAs were then diluted (1:10) from 400 to 0.4 nM per 100 μL of PBS+2% FBS, warmed to 37 °C, and added (100 μL) to the cells, bringing the total assay volume to 200 μL and the concentrations in the 200-0.2 nM range. A neat-PBS group served as the baseline control. Cells were stimulated at 37 °C for 20 min, followed by fixation at 1:1 (v/v) (BD Phosflow™ Fix Buffer I, 557870), a wash step, permeabilization with 500 μL (BD Phosflow™ Perm Buffer III, 558050) at −20 °C overnight, and 3 more washes with 1 mL each of PBS + 2% FBS in preparation for staining. Each well was resuspended in 100 μL of an antibody cocktail comprising CD3, CD4, CD8, and pSTAT5, detailed in Supplementary File S3, and stained at room temperature, away from light, for 45 min. Following two washes in PBS + 2% FBS, stained cells were acquired on a 5-laser Cytek Aurora Spectral Cytometer. pSTAT5 readouts were gated (lymphocytes → singlets → live → CD3 + pSTAT5), and median fluorescence intensities (MFIs) across all IL-2 SCA concentrations were extracted using CellEngine (Cell Carta, Montreal, QC, Canada) software and visualized with GraphPad Prism 10 (Supplemental File S3).

### 5.12. Crystallography

IL-18 and IL-2 SCAs were purified in 20 mM HEPES pH 7.5 and 150 mL NaCl and crystallized using sitting drop vapor diffusion at room temperature in 200 nl drops comprising 100 nl protein solution at the respective concentration and 100 nl reservoir solution. IL-2 SCA DR638 was concentrated to 65 mg/mL and crystallized with a reservoir solution comprising 10% (*v*/*v*) glycerol, 0.1 M HEPES pH 7.5, 30% (*v*/*v*) PEG 400, and 5% (*w*/*v*) PEG 3000. Crystals were frozen in liquid nitrogen without further addition of cryoprotectant. IL-18 SCA DR3085 was subjected to lysine methylation to aid crystallization using the Hampton Research Reductive Alkylation kit and crystallized at 25 mg/mL using a reservoir solution of 0.2 M lithium sulfate, 0.1 M Tris pH 8.5, and 1.26 M ammonium sulfate. Crystals were frozen in liquid nitrogen using 30% ethylene glycol as a cryoprotectant added to the reservoir solution. IL-18 SCA DR3087 crystallized against a reservoir comprising 0.1 M MES pH 6.0 and 1.6 M magnesium sulfate at a protein stock concentration of 35 mg/mL. Crystals were frozen in liquid nitrogen using 30% ethylene glycol as a cryoprotectant added to the reservoir solution. IL-18 SCA DR3097 was crystallized at 26 mg/mL using a reservoir comprising 3.6 M sodium formate and 10% (*v*/*v*) glycerol. Crystals were frozen in liquid nitrogen using 20% glycerol as a cryoprotectant added to the reservoir solution. X-ray diffraction data were collected at the Stanford Synchrotron Radiation Lightsource (SSRL) beamlines 12-1 (DR3085, DR3097, DR638) and 12-2 (DR3087) operating at a wavelength of 0.979 Å. Data were indexed in XDS [28], scaled with Aimless [29], and phases were determined using Phaser [30] via molecular replacement. VHH search models for the structures were based on PDB ID 6B20. Structures were refined in Refmac [31], and models were built using ISOLDE [32] as a UCSF ChimeraX [33] plugin and Coot [34]. Structures were visualized and analyzed in UCSF ChimeraX. X-ray data collection and refinement statistics are shown in Table 2.

### 5.13. DR3097 Disulfide Stapling, Stability Test, and Analysis

DR3097 disulfide staples were introduced through cysteine mutations between the following residues: S1: S7C-Q171C, S2: L112C-L174C, S3: L112C-A187C, S4: T114C-T177C, S5: T114C-A185C, S6: S116C-S160C, and S7: S116C-T179C. DR3097 and disulfide-stapled proteins were expressed and purified as described above. Disulfide formation was confirmed by LC/MS on an Agilent Q-tof instrument equipped with a Mabpac column equilibrated in 20% acetonitrile, 0.1% formic acid (20–80% acetonitrile elution gradient). Thermostability was monitored as NanoDSF, turbidity, and particle size as a function of a 1 °C/min temperature ramp were measured in triplicate at 1 mg/mL in PBS on a Nanotemper Panta instrument. Tm values were extracted from triplicate measurements with fit error sigma ≤ 0.05. Binding to IL-18Rα and IL-18Rβ receptors was analyzed by SPR. Data were collected on a Biacore T200 SPR system using an AHC kit with antibody immobilized on a CM5 SPR sensor chip. IL-18Rα and IL-18Rβ ECDs fused to human IgG1 Fc were captured as ligand; IL-18 SCAs were flown as analyte at indicated concentrations. Data were fitted using 1:1 binding kinetic models. For the stability test of DR3097, S4 and S5, the protein concentration was adjusted to 0.8 mg/mL, and samples were incubated for 0 days (0d), 3 days (3d), or 7 days (7d) at 60 °C. Protein precipitate was removed by centrifugation, and the soluble fraction was placed at 4 °C for further analysis. Downstream SEC analysis was performed using this soluble fraction diluted 1:1 in PBS without adjustment of protein concentration and using a Superdex 200 increase 10/300 GL column equilibrated in PBS at a 1 mL/min flow rate. Downstream SPR analysis was performed using a 1:2 dilution series starting from 400 nM for all S4 and S5 samples and the DR3097 control samples. Due to severe precipitation, DR3097 3d and 7d samples were not analyzed.

## Figures and Tables

**Table 1:** IL-18 and IL-2 SCA activity can be modulated through linker-length engineering. DR3085, DR3097, and DR3097 IL-18 SCA partial agonism can be modulated via length variation in a flexible inter-VHH linker. The table shows IFNγ maximum levels (ng/mL) and EC50 (nM) values for each variant extracted with a log agonist vs. response fit.

		Linker Length (Residues)					
		0	2	4	10	15	20
DR3085	EC50 (nM)	0.643	0.148	0.121	0.064	0.055	0.085
	Emax (ng/mL)	3.4	12.6	80.4	152.6	112.6	90.6
DR3087	EC50 (nM)	1.491	2.861	0.771	1.157		
	Emax (ng/mL)	109.9	90.8	162.5	86.7		
DR3097	EC50 (nM)	0.088	0.012	0.002	0.001	0.002	0.031
	Emax (ng/mL)	1.7	37.0	156.0	246.4	190.6	180.5

**Table 2:** X-ray data collection and refinement statistics (molecular replacement).

SCA Name PDB ID	DR638 9O99	DR3085 9O9C	DR3087 9O9A	DR3097 9O9B
**Data collection**				
Space group	P 21 21 21	P 65	C 1 2 1	P 3
Cell dimensions				
*a*, *b*, *c* (Å)	49.21, 63.13, 87.45	92.64, 92.64, 181.85	91.81, 172.66, 59.29	120.1, 120.1, 29.27
a, b, γ (°)	90, 90, 90	90, 90, 120	90, 90.01, 90	90, 90, 120
Resolution (Å) *	38.81–1.80 (1.84–1.80)	39.17–3.80 (4.25–3.80)	37.66–2.4 (2.49–2.4)	39.31–2.8 (2.95–2.80)
*R*_sym_ or *R*_merge_ *	0.067 (0.774)	0.34 (0.748)	0.055 (0.788)	0.112 (0.672)
*I*/s*I*	12.8 (3.1)	3.3 (1.6)	11.8 (1.6)	6.7 (1.6)
Completeness (%) *	99.6 (99.9)	99.2 (100.0)	95.6 (97.1)	96.3 (97.9)
Redundancy *	4.5 (4.5)	3.0 (3.1)	3.7 (3.7)	2.8 (2.8)
**Refinement**				
Resolution (Å)	1.8	3.80	2.4	2.8
No. reflections	24498	8208	32612	11206
*R*_work_/*R*_free_	0.180/0.211	0.238/0.291	0.241/0.280	0.246/0.283
No. nonhydrogen atoms	2088	3447	3533	3263
Protein	1820	3447	3486	3263
Ligand/ion	0	0	0	0
Water	268	0	47	0
*B*-factors				
Protein	26.6	87.7	74.9	64.4
Ligand/ion				
Water	40.1		58.1	
R.m.s. deviations				
Bond lengths (Å)	0.009	0.004	0.007	0.007
Bond angles (°)	1.767	1.283	1.718	1.331
Ramachandran				
Favored (%)	97.95	91.91	94.77	91.5
Allowed (%)	2.05	8.09	5.00	8.5
Outliers (%)	0	0	0.23	0

* Values in parentheses are for the highest-resolution shell.

## Data Availability

All the information used is available to the public. The data extracted from the studies and any other material used in the review are presented in the different sections of the work, in the annexes, or referenced in the bibliography.

## Supplementary Materials

The following supporting information can be downloaded at: https://www.mdpi.com/article/10.3390/antib14030074/s1. File S1: LCMS confirms disulfide bond formation for six out of seven engineered, stapled DR3097 SCAs. File S2: SEC-MALS of DR3097 anti-IL-18Rα and anti-IL-18Rβ mono-VHHs shows that they do not associate into dimers in solution. File S3: pSTAT5 measurements by flow cytometry.

## Abbreviations

AHC	Anti-Human Capture
CDRs	Complementary determining regions
CFA	Complete Freund’s Adjuvant
DLS	Dynamic light scattering
DSF	Differential scanning fluorimetry
ECD	Extracellular domain
IFA	Incomplete Freund’s Adjuvant
IFNγ	Interferon γ
HEK	Human embryonic kidney
IgG	Immunoglobulin G
IL-2	Interleukin 2
IL-2Rβ	Interleukin-2 receptor β
IL-2Rγ	Interleukin-2 receptor γ
IL-18	Interleukin 18
IL-18BP	IL-18 binding protein
IL-18Rα	Interleukin-18 receptor α
IL-18Rβ	Interleukin-18 receptor β
LCMS	Liquid chromatography-mass spectrometry
mAb	Monoclonal antibody
MSA	Mouse serum albumin
NF-kB	Nuclear factor kappa B
PBMC	Peripheral blood mononuclear cell
PBS	Phosphate-buffered saline
SCA	Surrogate cytokine agonist
scFv	Single-chain fragment variable
sdAbs	Single domain antibodies
SEAP	Secreted embryonic alkaline phosphatase
SEC	Size exclusion chromatography
SPR	Surface plasmon resonance
STAT	Signal transducer and activator of transcription
TIR	Toll/interleukin-1 receptor
Tm	Melting temperature
VHH	Variable heavy domain of Heavy chain
