# Supplementary material for: Structure-Guided Stapling of Dimeric Conformations and Linker Engineering Enhance Thermostability and Fine-Tune Activity of Bispecific VHH Cytokine Agonists
Source: Antibodies (Basel). 2025 Sep 1;14(3):74. doi: 10.3390/antib14030074 (PMC12452691; doi:10.3390/antib14030074)
Supplement: Supplementary file 1 [file antibodies-14-00074-s001.zip › 250716 File S1.pptx]

## Slide 1
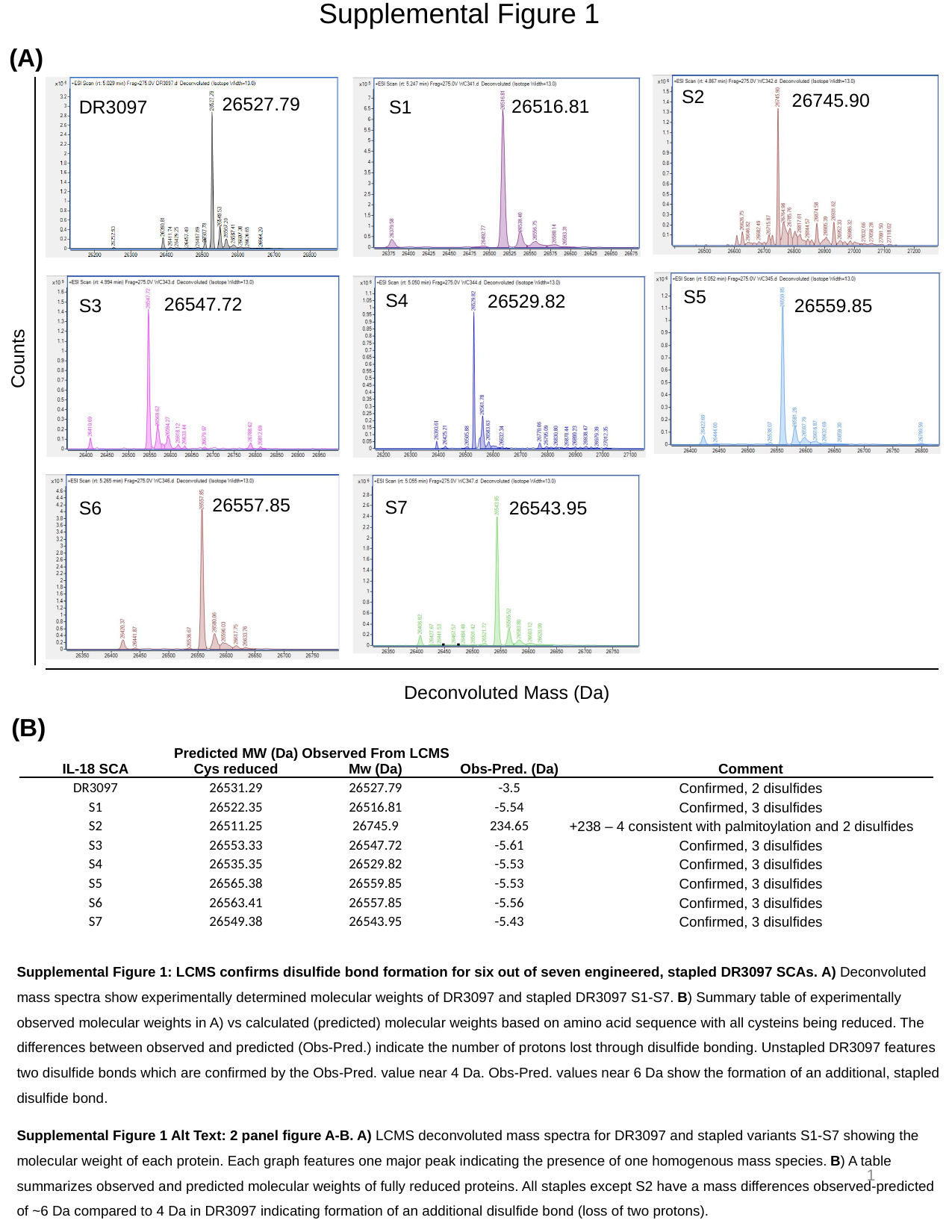

Supplemental Figure 1
(A)
S2
26745.90
26527.79
26516.81
DR3097
S1
S5
S4
26529.82
26547.72
S3
26559.85
Counts
26557.85
S7
S6
26543.95
Deconvoluted Mass (Da)
(B)
| IL-18 SCA | Predicted MW (Da) Cys reduced | Observed From LCMS Mw (Da) | Obs-Pred. (Da) | Comment |
| --- | --- | --- | --- | --- |
| DR3097 | 26531.29 | 26527.79 | -3.5 | Confirmed, 2 disulfides |
| S1 | 26522.35 | 26516.81 | -5.54 | Confirmed, 3 disulfides |
| S2 | 26511.25 | 26745.9 | 234.65 | +238 – 4 consistent with palmitoylation and 2 disulfides |
| S3 | 26553.33 | 26547.72 | -5.61 | Confirmed, 3 disulfides |
| S4 | 26535.35 | 26529.82 | -5.53 | Confirmed, 3 disulfides |
| S5 | 26565.38 | 26559.85 | -5.53 | Confirmed, 3 disulfides |
| S6 | 26563.41 | 26557.85 | -5.56 | Confirmed, 3 disulfides |
| S7 | 26549.38 | 26543.95 | -5.43 | Confirmed, 3 disulfides |
Supplemental Figure 1: LCMS confirms disulfide bond formation for six out of seven engineered, stapled DR3097 SCAs. A) Deconvoluted mass spectra show experimentally determined molecular weights of DR3097 and stapled DR3097 S1-S7. B) Summary table of experimentally observed molecular weights in A) vs calculated (predicted) molecular weights based on amino acid sequence with all cysteins being reduced. The differences between observed and predicted (Obs-Pred.) indicate the number of protons lost through disulfide bonding. Unstapled DR3097 features two disulfide bonds which are confirmed by the Obs-Pred. value near 4 Da. Obs-Pred. values near 6 Da show the formation of an additional, stapled disulfide bond.
Supplemental Figure 1 Alt Text: 2 panel figure A-B. A) LCMS deconvoluted mass spectra for DR3097 and stapled variants S1-S7 showing the molecular weight of each protein. Each graph features one major peak indicating the presence of one homogenous mass species. B) A table summarizes observed and predicted molecular weights of fully reduced proteins. All staples except S2 have a mass differences observed-predicted of ~6 Da compared to 4 Da in DR3097 indicating formation of an additional disulfide bond (loss of two protons).
1
