# Supplementary material for: Structure-Guided Stapling of Dimeric Conformations and Linker Engineering Enhance Thermostability and Fine-Tune Activity of Bispecific VHH Cytokine Agonists
Source: Antibodies (Basel). 2025 Sep 1;14(3):74. doi: 10.3390/antib14030074 (PMC12452691; doi:10.3390/antib14030074)
Supplement: Supplementary file 1 [file antibodies-14-00074-s001.zip › 250804 File S3.pptx]

## Slide 1
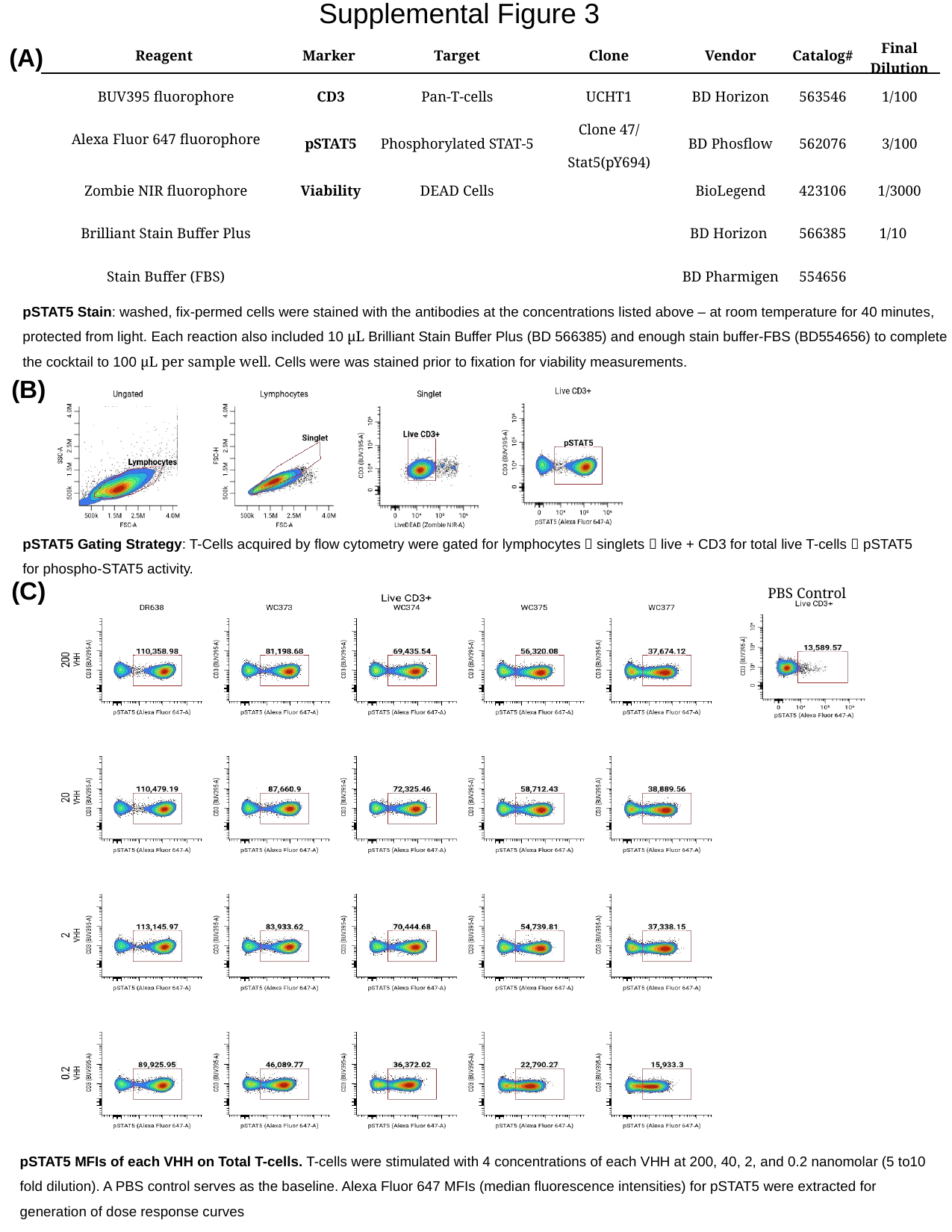

Supplemental Figure 3
| Reagent | Marker | Target | Target | Clone | Clone | Vendor | Catalog# | Final Dilution | Volume (µL) |
| --- | --- | --- | --- | --- | --- | --- | --- | --- | --- |
| BUV395 fluorophore | CD3 | Pan-T-cells | Pan-T-cells | UCHT1 | UCHT1 | BD Horizon | 563546 | 1/100 | 1 |
| Alexa Fluor 647 fluorophore | pSTAT5 | Phosphorylated STAT-5 | Phosphorylated STAT-5 | Clone 47/ Stat5(pY694) | Clone 47/ Stat5(pY694) | BD Phosflow | 562076 | 3/100 | 3 |
| Zombie NIR fluorophore | Viability | DEAD Cells | DEAD Cells | | | BioLegend | 423106 | 1/3000 | |
| Brilliant Stain Buffer Plus | | | | | | BD Horizon | 566385 | 1/10 | |
| Stain Buffer (FBS) | | | | | | BD Pharmigen | 554656 | | |
(A)
pSTAT5 Stain: washed, fix-permed cells were stained with the antibodies at the concentrations listed above – at room temperature for 40 minutes, protected from light. Each reaction also included 10 µL Brilliant Stain Buffer Plus (BD 566385) and enough stain buffer-FBS (BD554656) to complete the cocktail to 100 µL per sample well. Cells were was stained prior to fixation for viability measurements.
(B)
pSTAT5 Gating Strategy: T-Cells acquired by flow cytometry were gated for lymphocytes  singlets  live + CD3 for total live T-cells  pSTAT5 for phospho-STAT5 activity.
(C)
PBS Control
pSTAT5 MFIs of each VHH on Total T-cells. T-cells were stimulated with 4 concentrations of each VHH at 200, 40, 2, and 0.2 nanomolar (5 to10 fold dilution). A PBS control serves as the baseline. Alexa Fluor 647 MFIs (median fluorescence intensities) for pSTAT5 were extracted for generation of dose response curves
