# Supplementary material for: Structure-Guided Stapling of Dimeric Conformations and Linker Engineering Enhance Thermostability and Fine-Tune Activity of Bispecific VHH Cytokine Agonists
Source: Antibodies (Basel). 2025 Sep 1;14(3):74. doi: 10.3390/antib14030074 (PMC12452691; doi:10.3390/antib14030074)
Supplement: Supplementary file 1 [file antibodies-14-00074-s001.zip › 250716 File S2.pptx]

## Slide 1
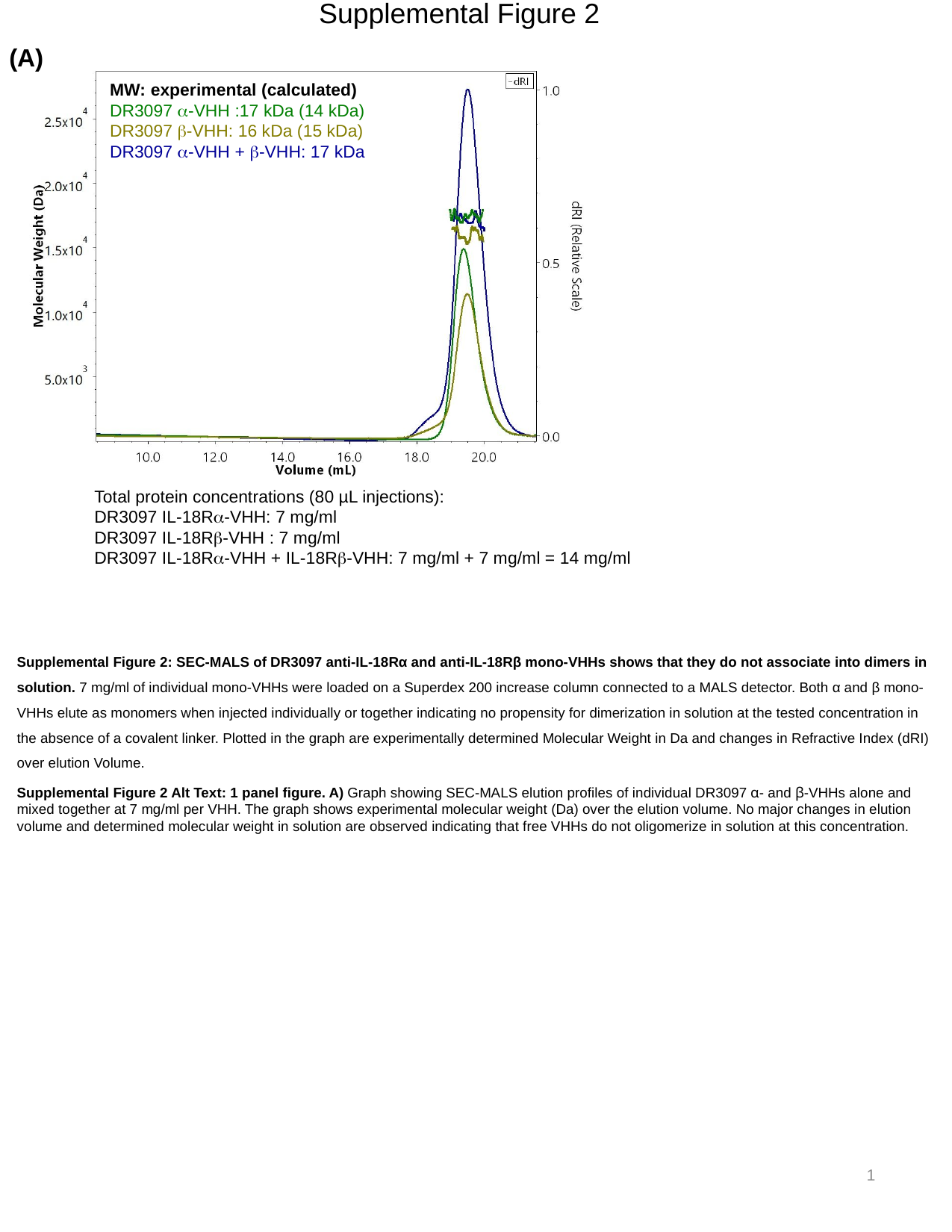

Supplemental Figure 2
(A)
MW: experimental (calculated)
DR3097 a-VHH :17 kDa (14 kDa)
DR3097 b-VHH: 16 kDa (15 kDa)
DR3097 a-VHH + b-VHH: 17 kDa
Total protein concentrations (80 µL injections):
DR3097 IL-18Ra-VHH: 7 mg/ml
DR3097 IL-18Rb-VHH : 7 mg/ml
DR3097 IL-18Ra-VHH + IL-18Rb-VHH: 7 mg/ml + 7 mg/ml = 14 mg/ml
Supplemental Figure 2: SEC-MALS of DR3097 anti-IL-18Rα and anti-IL-18Rβ mono-VHHs shows that they do not associate into dimers in solution. 7 mg/ml of individual mono-VHHs were loaded on a Superdex 200 increase column connected to a MALS detector. Both α and β mono-VHHs elute as monomers when injected individually or together indicating no propensity for dimerization in solution at the tested concentration in the absence of a covalent linker. Plotted in the graph are experimentally determined Molecular Weight in Da and changes in Refractive Index (dRI) over elution Volume.
Supplemental Figure 2 Alt Text: 1 panel figure. A) Graph showing SEC-MALS elution profiles of individual DR3097 α- and β-VHHs alone and mixed together at 7 mg/ml per VHH. The graph shows experimental molecular weight (Da) over the elution volume. No major changes in elution volume and determined molecular weight in solution are observed indicating that free VHHs do not oligomerize in solution at this concentration.
1
